# Evaluation of Uric Acid as a Prognostic Blood-Based Marker in a Large Cohort of Pancreatic Cancer Patients

**DOI:** 10.1371/journal.pone.0104730

**Published:** 2014-08-18

**Authors:** Michael Stotz, Joanna Szkandera, Julia Seidel, Tatjana Stojakovic, Hellmut Samonigg, Daniel Reitz, Thomas Gary, Peter Kornprat, Renate Schaberl-Moser, Gerald Hoefler, Armin Gerger, Martin Pichler

**Affiliations:** 1 Division of Clinical Oncology, Department of Medicine, Medical University of Graz, Graz, Austria; 2 Clinical Institute of Medical and Chemical Laboratory Diagnostics, Medical University of Graz, Graz, Austria; 3 Division of Angiology, Department of Medicine, Medical University of Graz, Graz, Austria; 4 Division of General Surgery, Department of Surgery, Medical University of Graz, Graz, Austria; 5 Institute of Pathology, Medical University of Graz, Graz, Austria; 6 Research Unit Genetic Epidemiology and Pharmacogenetics, Division of Clinical Oncology, Department of Medicine, Medical University of Graz, Graz, Austria; 7 Department of Experimental Therapeutics, University of Texas MD Anderson Cancer Center, Houston, Texas, United States of America; Baylor College of Medicine, United States of America

## Abstract

**Background:**

Recently, chemical blood parameters gain more attraction as potential prognostic parameters in pancreatic cancer (PC). In the present study we investigated the prognostic relevance of the uric acid (UA) level in blood plasma at the time of diagnosis for overall survival (OS) in a large cohort of patients with PC.

**Patients and Methods:**

Data from 466 consecutive patients with ductal adenocarcinoma of the pancreas were evaluated retrospectively. Overall survival (OS) was analysed using the Kaplan-Meier method. To further evaluate the prognostic significance of the UA level, univariate and multivariate Cox regression models were calculated.

**Results:**

None of the clinicopathological parameters (tumour grade, clinical stage, age, CA19-9 level, Karnofski Index (KI) or surgical resection) except gender was associated with UA level. In univariate analysis we observed the elevated UA level (<5.1 versus ≥5.1 mg/dl, *p* = 0.017) as poor prognostic factor for OS. In the multivariate analysis that included age, gender, tumour grade, tumour stage, surgical resection, CA19-9 level, the KI and UA level we confirmed the UA level as independent prognostic factor for OS (HR = 1.373%; CI = 1.077–1.751; *p* = 0.011).

**Conclusion:**

In conclusion, we identified the UA level at time of diagnosis as an independent prognostic factor in PC patients. Our results indicate that the UA level might represent a novel and useful marker for patient stratification in PC management.

## Introduction

During the last years incidence and mortality of pancreatic cancer (PC) as the second most common cause of cancer related death among all gastrointestinal malignancies has only slightly changed [1], [2]. In more detail, PC's mortality after R0 resection, which is indeed the only measurement of cure, still remains poor, regarding the 5-year survival rate of about 25 percent, falling to only 8 percent after 10 years [2]. However, at time of diagnosis less than 20 percent of patients are resectable [3]. The dismal prognosis of PC patients results from the long symptom free time resulting in pronounced local tumour progression and establishment of distant metastases. Despite testing of neoadjuvant chemotherapy protocols in order to achieve higher resectability, this therapeutic modality is not proven useful in terms of survival yet [4], [5]. So far, several histopathological prognostic factors, such as tumour size, histological subtype or grade, vascular invasion and lymph node metastases are known [6]–[10]. However, the prognostic markers being on hand are suboptimal, due to the fact that invasive approach to the tumour is needed to receive the majority of these prognostic factors. More easily available and low cost parameters might be more practicable and more patient-friendly [11]–[13].

Uric acid is a product of the metabolic breakdown of purine nucleotides and also indicates a higher turnover of nucleic acids in cellular elements. It develops through rapid catabolism of purine-containing nucleic acids from tumour cells [14]. UA levels could recently be shown to be prognostically relevant in nasopharyngeal carcinoma patients and colon cancer patients [15], [16]. Currently, there is no data in PC regarding the UA as a prognostic marker.

The aim of the present study was to investigate the clinical effect of the UA in blood plasma at the time of diagnosis for overall survival (OS) in a large cohort of patients with PC.

## Materials and Methods

This retrospective analysis included data from 466 consecutive patients who were treated at the Division of Clinical Oncology, Medical University of Graz, between 2004 and 2012. All patients had histological confirmed ductal adenocarcinoma of the pancreas and available UA levels at the time of diagnosis. All clinico-pathological data were retrieved from medical records at the Division of Clinical Oncology, as well as from pathology records from the Institute of Pathology at the same institution. Since the TNM classification system for PC changed during the study period, tumour stages were uniformly adjusted according to the 7^th^ edition of this system. Other documented clinico-pathological parameters included administration of chemotherapy with gemcitabine, gender and age. The UA levels were obtained by exploration within 1 to 3 days before histological proven diagnosis. Follow-up evaluations were performed every three months within the first three years, six months for five years and annually thereafter for curative resected tumour stages. Follow-up investigations included clinical check-up, laboratory including CEA and CA 19-9, and radiological assessment. For deceased patients, dates of death were obtained from the central registry of the Austrian Bureau of Statistics. Patient records were anonymized and de-identified prior to analysis. The study was approved by the local ethical committee of the Medical University of Graz (No. 26-196 ex 13/14).

### Statistical analyses

Overall survival was defined as the time (in months) from date of surgery or date of histological proven diagnosis to death of any cause. The optimal cut off level for the CA19-9 and UA to differentiate between survival and death was determined by receiver operating curve analysis, as previously described [17]. The association between the UA and clinico-pathological parameters was evaluated by non-parametric tests (Mann-Whitney U and chi square test). Patients' clinical endpoint was calculated using the Kaplan-Meier method and compared by the log rank test. Backward stepwise multivariate Cox proportion analysis was performed to determine the influence of different clinico-pathological parameters and UA on OS. Hazard ratios (HRs) estimated from the Cox analyses were reported as relative risks with corresponding 95% confidence intervals. All statistical analyses were performed using the Statistical Package for Social Sciences version 21.0 (SPSS Inc., Chicago, IL, USA). A two-sided *p*<0.05 was considered statistically significant.

## Results

Overall, 252 male and 214 female patients with PC were included in the study cohort. The mean age at diagnosis was 64.6±10.4 years. The median follow-up period was 36 months (range, 0–162 months). Median survival was 7 months (range, 0–59 months) and 292 (92.4%) patients had died by their most recent follow-up visit. The tumour stage was defined as stage I or II in 108 patients (23.2%), stage III in 31 patients (6.7%) and stage IV in 327 patients (70.2%). Baseline patient characteristics and tumour biological factors are shown in Table 1. Applying receiver operating curve analysis, we calculated an optimal cut-off level for the UA as 5.1 mg/dl. (normal range 3,4–7,0 mg/dl).

**Table 1:** The relation between clinico-pathological parameters and the pretreatment uric acid level of patients with pancreatic adenocarcinoma (*n* = 466).

Characteristics	UA level<5.1 mg/dl	UA level ≥5.1 mg/dl	*p*-value
**Gender**			
Female	163	51	<0.001
Male	141	111	
**Surgical resection**			
No	210	123	0.073
Yes	94	39	
**Tumour stage**			
I–II	75	33	0.445
III	18	13	
IV	211	116	
**Tumour grade**			
G1+G2	190	97	0.324
G3+G4	114	65	
**CA19-9 elevated**			
<750 U/l	128	72	0.311
>750 U/l	135	67	
**Karnofski Index (KI)**			
≤80	183	102	
>80	120	60	0.661
unknown	1	0	

In our study cohort none of the clinico-pathological parameters (tumour grade, clinical stage, age, CA19-9 level, Karnofski Index (KI) or surgical resection) except gender was associated with UA level (Table 1). To investigate whether the UA and other clinico-pathological factors are associated with clinical outcome of PC patients, univariate and multivariate Cox proportional models for OS were calculated.

**Figure 1:**
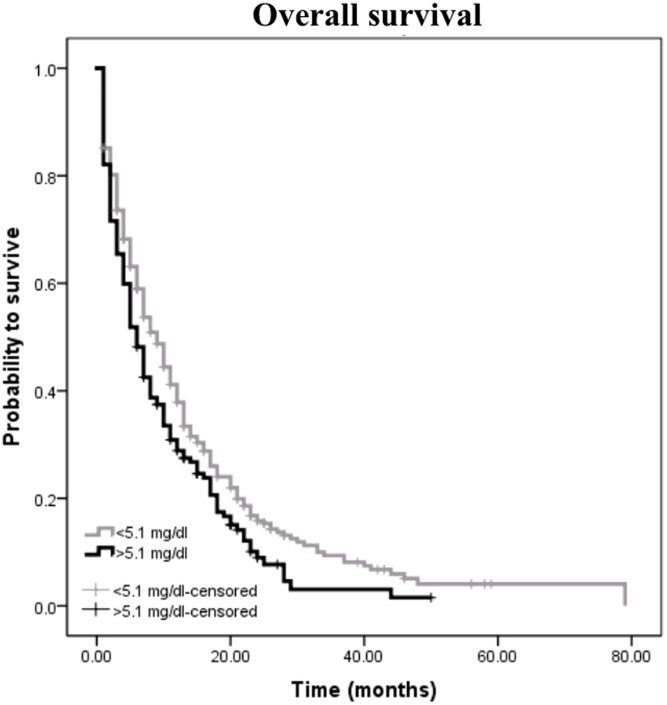
Kaplan-Meier curve for overall survival regarding UA level <5.1 mg/dl versus UA level ≥5.1 mg/dl in the whole cohort of patients (*p*<0.013).

Among the 466 PC patients, death occured in 256 of 304 (84.2%) patients with a UA level<5.1 and in 143 of 162 (88.3%) patients with a UA level≥5.1 (*p* = 0.013). Figure 1 shows the Kaplan-Meier curves for OS and reveals that an UA level higher than 5.1 is a consistent factor for poor prognosis in PC patients (*p* = 0.013, log-rank test).

Univariate analysis identified a high tumour stage (stage I, II, III versus IV, *p*<0.001), a high tumour grade (G1, G2 versus G3, G4, *p* = 0.013), surgical resection (surgical resection versus no surgical resection, *p*<0.001), the CA19-9 level (<750 U/l versus>750 Ul *p*<0.001), the KI (80%≤versus>80%, *p* = 0.047) and the UA level (<5.1 versus ≥5.1 mg/dl, *p* = 0.017) as poor prognostic factors for OS in this study cohort. Gender was not significantly associated with clinical outcome (Table 2). The UA level correlated with the creatinine levels (R = 0.435, p<0.001, Spearman correlation) and inversely correlated with the calculated glomerular filtration rate (R = −0.353, p<0.001, Spearman correlation). Glomerular filtration rate was calculated using the CKD-EPI formula. However, both of these renal functions variables were not associated with survival in univariate analysis in our study cohort (for creatinine levels hazard ratio: 1.43 (95%CI 0.93–2.18), p = 0.096, for glomerular filtration rate hazard ratio: 0.998 (95%CI 0.994–1.01), p = 0.171).

**Table 2:** Univariate and multivariate Cox proportional analysis regarding overall survival.

Parameter	Univariate analysis		Multivariate analysis	
	HR (95% CI)	*p*-value	HR (95% CI)	*p*-value
**Gender**				
Female	1 (reference)	0.198	1 (reference)	0.73
Male	1.14 (0.93–1.39)		0.96 (0.76–1.21)	
**Tumour stage**				
Stage I–II	1 (reference)		1 (reference)	
Stage III	3.00 (1.85–4.88)	<0.001	2.95 (1.55–5.62)	0.001
Stage IV	3.88 (2.93–5.13)	<0.001	4.02 (2.21–7.32)	<0.001
**Tumour grade**				
G1+G2	1 (reference)	0.013	1 (reference)	0.001
G3+G4	1.29 (1.06–1.58)		1.45 (1.16–1.82)	
**CA19-9 elevated**				
No	1 (reference)	<0.001	1 (reference)	<0.001
Yes	1.98 (1.60–2.47)		1.54 (1.23–1.93)	
**UA level**				
<5.1	1 (reference)	0.017	1 (reference)	0.011
≥5.1	1.28 (1.05–1.58)		1.37 (1.08–1.75)	
**Karnofsky Index**				
≤80	1 (reference)	0.047	1 (reference)	0.634
>80	0.93 (0.85–0.99)		0.98 (0.88–1.08)	
**Surgical resection**				
No	1 (reference)	<0.001	1 (reference)	0.713
Yes	0.33 (0.26–0.42)		1.10 (0.65–1.87)	

To determine the independent prognostic value of UA level for OS, a multivariate analysis using a Cox proportional hazard model was performed. In the multivariate analysis that included age, gender, tumour grade, tumour stage, surgical resection, CA19-9 level, the KI and UA level we identified tumour grade, tumour stage, the CA19-9 level and the UA level (HR = 1.373%; CI = 1.077–1.751; *p* = 0.011) as independent prognostic factors for OS (Table 2).

## Discussion

Regarding the moderate progress in tumour-specific therapy in PC management of PC patients still stays a great challenge. The initial tumour stage and possibility of curative resection are ongoing key factors for further oncological therapy decision. However, the majority of PC patients are treated in a palliative setting, owing to failure of surgical resection at the time of diagnosis. Recently, the role of biomarkers have been intensively investigated, with, overall, very promising results [11], [13], [18]. In order to obtain more prognostic information than pathological tissue examination or traditional clinico-pathological variables in PC, we examined a large cohort of patients with PC and found a significant association between the level of UA in the blood plasma and OS. To the best of our knowledge this is the first study investigating the role of pretreatment UA as prognostic marker in PC.

UA is the final end product of purine metabolism and extracted in urine. The cytosolic enzyme adenosine deaminase catalyzes the hytrolytic deamination of adenosine to inosine, which can be deribosylated by purine nucleoside phosphorylase, converting it to hypoxanthine. Xanthine oxidase then forms UA from hypoxanthine as well as from xanthine. Various diseases such as diabetes, cardiovascular disease or, in general, metabolic syndrom go along with elevated levels of UA [19]. There are two main reasons for elevated UA concentrations – as a result of increased production of UA or reduction of UA secretion through the kidney, respectively. The antioxidantial potential of UA itself has recently become better investigated due to its role of being capable of protecting against free-radical oxidative damage [20], [21]. Reactive oxygen radicals play a major role in the initiation of a tumourous disease by increasing the mutation rate in cells, leading to a high oncogenic potential [22], [23]. In tumour cells, oxidative stress has been associated with DNA damage, cellular adhesion and migration capacity, proliferation and the regulation of cell survival or death [24], [25]. UA also induces proliferation, inflammation and is involved in intracellular redox dependent mechanisms, but the definitive function in cell and tissue have not been fully explained yet [26]. Moreover, a high plasma UA level, despite various other reasons, may occur in patients as a result of increased purine metabolism by xanthine oxidase, as a consequence of tumour cell breakdown. Elevated cell turnover, as a result of cellular injury, could indeed be identified as mechanism for raised production of UA in cancer patients. Damaged cells degrade their RNA and DNA, resulting in a rapidly increasing concentration of UA [27]–[29]. Shi et al. showed UA being released by dying cells together with their antigens, thereby stimulating dendritic cells to mature, resulting in stimulation of the immune system – especially CD8+ T-Lymphocytes [20], [21]. Lymphocytes play a crucial role in tumour defense by inducing cytotoxic cell death and inhibiting tumour cell proliferation and migration [30], [31]. Regarding these findings, an elevated level of UA should be associated with better prognosis, which could indeed be found by Dziaman et al. showing elevated levels of UA going along with longer survival time in colorectal cancer patients [16]. However, this is in contrast with our findings, as in our study an elevated pre-treatment UA level was associated with a shorter OS. This discrepancy could partly be explained by the findings of Xu et al., who found accumulation of UA and MAP (mitogen-activated protein) kinase activation resulting in increased MICA/B expression after chemotherapy [32]. Regarding the findings of Xu et al., it is imaginable, that because of elevated UA levels resulting of undergoing tumour cells (hypoxia, tumour microenvironment acidosis) or non tumourous reasons for pretreatment hyperuricaemia (gout, metabolic syndrom) inflammatory anti-tumoural reaction is weaker. This would go along with findings of Fini et al., who could show hyperuricaemia to be associated with elevated tumour incidence and mortality [33]. Shin et al. demonstrated, that high serum UA levels were significantly and independently associated with short survival time in terminally ill cancer patients [34]. However, we could rather speculate about the pathophysiological background underlying these prognostic findings of UA level at time of diagnosis because available data are conflicting.

Dhankhar et al. found UA as well as adenosine deaminase elevated in patients with head and neck carcinoma and the levels were observed to rise in higher stages [35]. Seeing UA as a marker simply reflecting cellular proliferation, increased cell turnover and, thus, purine catabolism, the UA level showed to be associated with higher stage or grade. In fact, in our study we could not observe this. Nonetheless, we could show that an elevated UA level goes along with a significantly shorter cancer survival time.

Based on our findings, the non-invasive measurement of the UA level at the time of PC diagnosis seems to be a new marker for individual patient's risk assessment. In comparison to other tumour entities the prognostic value in daily clinical practice of the uric acid is clearly limited by its relatively low hazard ratio and as a result of poor prognosis of PC in general. However, there might be some clinical scenarios where additional prognostic parameters are helpful in refined risk stratification. An additional risk assessment variable can help for better individual patients counselling. Second, as novel more effective regimens become available for PC patients including FOLFIRINOX regimen or gemcitabine/nab-paclitaxel, an additional prognostic marker might help to decide which patient should receive a more aggressive but also more toxic treatment [36], [37]. Moreover, patient stratification according to novel prognostic factors can help to avoid selection bias in clinical trials, where misbalance in (yet unknown) prognostic variables can lead to misinterpretation of treatment results.

The strength of this study is the large sample size and the long follow-up period. Additionally, the UA level provides an easily and generally available, low price biomarker. Nevertheless, because of the retrospective design of the study, we cannot fully exclude a selection bias in our study cohort. Our study indicates for the first time that a high UA level is a negative prognostic marker in PC patients.
